# Is a positive intracutaneous test induced by penicillin mediated by histamine? A cutaneous microdialysis study in penicillin-allergic patients

**DOI:** 10.1186/s13601-017-0179-2

**Published:** 2017-11-17

**Authors:** Line K. Tannert, Sidsel Falkencrone, Charlotte G. Mortz, Carsten Bindslev-Jensen, Per Stahl Skov

**Affiliations:** 1 Department of Dermatology and Allergy Center, Odense Research Center for Anaphylaxis, Odense University Hospital, Kløvervænget 15, 5000 Odense C, Denmark; 2 Reflab®, Copenhagen, Denmark

**Keywords:** Histamine release, Penicillin-allergy, Penicillin challenge, Penicillin intracutaneous test, Skin microdialysis

## Abstract

**Background:**

Diagnostic workup of penicillin allergy comprises skin testing with penicillins, and patients are deemed allergic if skin test is positive. However, the literature suggests that skin test-positive patients may be challenge-negative, indicating that the skin test may be falsely positive.

**Objective:**

To investigate real-time histamine release from a positive intracutaneous test induced by penicillin in patients with positive and negative challenges to penicillin.

**Methods:**

Skin microdialysis was performed in 21 penicillin-allergic patients with positive skin test, 13 non-allergic volunteers serving as negative controls, and 7 grass pollen-allergic patients serving as positive controls. Histamine was measured by microdialysis after skin test with penicillin/grass/NaCl. Penicillin challenge was subsequently performed in 12 of the patients.

**Results:**

Only 10/21 patients (47.6%) were skin test positive at microdialysis. During microdialysis 13 single intracutaneous tests were positive and histamine was detected in 4/13 occurring in four challenge positive patients. Thirteen/21 patients (61.9%) were deemed allergic to penicillin; eight had positive skin test. Two patients with positive skin test were challenge negative. In grass pollen allergic patients, 7/7 had a positive intracutaneous test to grass and all released histamine in the wheals. All 13 negative controls had negative intracutaneous test to penicillin and no histamine release.

**Conclusion:**

Histamine was only detected in the minority of positive intracutaneous tests with penicillin in penicillin-allergic patients. Other mediators may be involved.

## Background

Identification of penicillin allergy is important, and the diagnostic work-up of patients with suspected penicillin allergy consists of several steps according to guidelines [1–3]. One step is skin testing, and if either the skin prick test (SPT) or the intracutaneous test (ICT) is positive, the patient is deemed to have a life-long allergy to penicillin. Positive skin testing is considered reliable for the diagnosis of penicillin allergy, but we recently demonstrated that 60% of patients with a positive skin test were challenge-negative to the culprit penicillin [4]. A few other studies also reported negative challenges in patients with a positive skin test [5–12].

A positive SPT or ICT to an allergen is generally accepted to be due to histamine that is released in an IgE-mediated reaction from the skin mast cells, although these cells contain a large number of other mediators and are considered to be the orchestrating cells in initiation and dissemination of the allergic reaction [13]. Previous skin microdialysis studies have confirmed histamine release in wheals induced by grass in pollen-allergic patients [14]. Histamine release in wheals has also been demonstrated in non-IgE mediated reactions such as ice cube challenge in cold urticaria patients [15].

The aim of this study was to use skin microdialysis to investigate histamine release in patients with positive intracutaneous test to penicillin, and to compare the results with challenge outcome.

## Methods

### Participants

The following groups of patients and controls were included in the microdialysis study (Table 1).

**Table 1:** Demographic data and result of skin test, s-IgE, microdialysis and challenge in patients with a previous positive ICT to penicillin

Patient	Sex	Age	Initial reaction		S-IgE				ICT previously positive	ICT positive	Histamine in eluate	Challenge		Time interval (months)	
			Symptoms	Culprit drug	Pen V	Pen G	AMP	AX				Symptoms	Drug	Initial reaction → microdialysis	First ICT → microdialysis
1	M	64	Ana	MC	< 0.35	< 0.35	< 0.35	< 0.35	MC	MC	+	ND (Ana)	−	2	0.25
2	F	46	Ana	Pen V	< 0.35	< 0.35	< 0.35	< 0.35	BP	BP	+	ND (Ana)	−	6	4
3	F	46	Urt	Pen V	1.61	1.65	1.25	< 0.35	AMP, BP	AMP	−	ND (SRI)	−	91	84
4	M	58	Ana	DX	< 0.35	< 0.35	< 0.35	< 0.35	BP, DX, AMP, MC*	−	−	Positive (Urt)	DX	27	26
5	F	60	Urt + Ang	Pen V	< 0.35	< 0.35	< 0.35	< 0.35	BP, AMP*	−	−	Positive (Urt)	Pen V	90	84
6	F	23	Urt	Pen V	< 0.35	< 0.35	< 0.35	< 0.35	AX	−	−	Positive (Urt + Ang)	Pen V	5	0.5
7	F	58	Ana	Pen V	2.85	< 0.35	< 0.35	< 0.35	BP*	−	−	Positive (Urt)	Pen V	101	95
8	F	51	Urt	Pen V	2.43	< 0.35	< 0.35	< 0.35	BP, AMP, AX	BP, AMP, AX	−	Positive (Urt)	Pen V	3	0.5
9	F	50	Urt	AX + Cla	< 0.35	< 0.35	< 0.35	< 0.35	AX	AX	+	Positive (Urt)	AX + Cla	11	1
10	F	50	Urt	Pen V	< 0.35	< 0.35	< 0.35	< 0.35	BP	BP	+	Positive (Urt)	Pen V	1	0.25
11	F	63	Erythema	MC	< 0.35	< 0.35	< 0.35	< 0.35	MC	MC	−	ND (DPI)	−	4	3
12	F	39	Uns. rash	DX	< 0.35	< 0.35	< 0.35	< 0.35	DX	−	−	ND (DPI)	−	17	4
13	F	70	Uns. rash	Pen V	< 0.35	< 0.35	< 0.35	< 0.35	BP, MC	BP, AMP	−	ND (DPI)	−	240	0.5
14	F	46	Urt	Pen V	< 0.35	< 0.35	< 0.35	< 0.35	BP	−	−	Negative	Pen V	29	24
15	M	54	Urt	Pen V	< 0.35	< 0.35	< 0.35	< 0.35	BP	BP	−	Negative	Pen V	18	6
16	F	58	Urt	MC	< 0.35	< 0.35	1.64	1.19	BP, AMP, MC	−	−	Negative	MC + AMP	7	5
17	M	42	Uns. rash	AX	< 0.35	< 0.35	< 0.35	< 0.35	AX	−	−	Negative	AX	12	0.25
18	F	46	Urt	Pen V	< 0.35	< 0.35	< 0.35	< 0.35	AX	BP	−	Negative	Pen V	9	1
19	M	48	Ana	Pen V	2.50	< 0.35	< 0.35	< 0.35	BP, AMP	−	−	ND (refused)	−	40	39
20	M	55	Ana	Pen V	< 0.35	< 0.35	< 0.35	< 0.35	BP*	−	−	ND (refused)	−	82	79
21	F	45	Urt	Pen V	< 0.35	< 0.35	< 0.35	< 0.35	BP, AMP	−	−	ND (refused)	−	33	32

Age refers to patients’ age at the day of microdialysis. Challenge refers to challenge with penicillin performed after microdialysis. * Not skin tested with amoxicillin because it was not available at the time of testing. Patient 16 was challenged at two separate occasions

*Pen V* penicillin V, *Pen G* penicillin G, *AMP* ampicillin, *AX* amoxicillin, *MC* mecillinam, *Ana* recent anaphylaxis, *DPI* delayed positive ICT, *SRI* systemic reaction during ICT, *Urt* urticaria, *Ang* angioedema, *ND* not done

- Patients (*n* = 21) with a case history of allergic reaction to a penicillin and positive intracutaneous test to at least one type of penicillin; 6 men and 15 women, mean age 51 years (range 23–70 years). Of the 21 patients, 5 also had low levels of specific IgE to one or more penicillin (s-IgE). Median time interval between initial reaction and inclusion in the study was 40 months (range 1–240 months). Two of the patients had a case history of a non-immediate reaction occurring > 1 h after last administration of penicillin (Patient 12 and 13, Table 1). Two patients (Patient 8 and 17, Table 1) could not remember the exact timing from administration until reaction, and the remaining 17 had immediate reactions (occurring < 1 h after last penicillin administration).
- Healthy volunteers (*n* = 13) without any allergic reactions to penicillin treatment; 2 men and 11 women, mean age 45 years (range 27–62 years), served as negative controls.
- Grass pollen-allergic patients (*n* = 7); 3 men and 4 women, mean age 35 years (range 27–51 years), with a positive skin prick test and s-IgE to grass, suffering from rhinoconjunctivitis during the grass pollen season, and serving as positive “classical allergic” controls.

### Skin testing

An intracutaneous test with penicillin was performed during microdialysis with the penicillin(s) previously shown to induce a positive test. The following concentrations were used: benzylpenicillin 20 mg/mL, amoxicillin 20 mg/mL, ampicillin 20 mg/mL, dicloxacillin 1 mg/mL, and mecillinam 4 mg/mL both in patients and controls. Except for benzylpenicillin, concentrations were according to European Network for Drug Allergy (ENDA) guidelines [16].

During microdialysis, ICT was performed with the penicillin(s) that was positive at first ICT. None of the patients were tested with the major or minor determinants, PPL and MDM, during microdialysis. Skin testing with these reagents was not part of the routine testing at the Allergy Center, because Hjortlund et al. demonstrated that all patients with a positive PPL or MDM were also positive to benzylpenicillin [17]. However, 5/21 patients had a previous PPL and MDM skin test; one patient was positive to both but concomitantly positive to benzylpenicillin. Controls were tested with the same types of penicillin as the patients.

Intracutaneous test with grass was performed with Phleum pratense extract in a dilution of 1000 SQ-U/mL (ALK-Abello, Hørsholm, Denmark).

The non-IgE dependent histamine releaser, codeine (codeine phosphate 1 mg/mL RefLab^®^, Copenhagen, Denmark) was used as positive control to demonstrate releasability of histamine from skin mast cells and to release residual histamine after a positive ICT induced by penicillin or grass. Intracutaneous test with isotonic saline 0.9% was the negative control in all participants.

ICT was always performed by injecting 50 µL, and reactions were considered positive if the wheal size diameter was 3 mm larger than the initial bleb. ICT was read after 20 min, according to ENDA guidelines [2].

Regarding interpretation of the ICTs, all tests with penicillin in the group of 21 patients were assessed blinded with photographs of the reactions by three independent consultants with experience in skin testing. Evaluations were in full compliance with the investigators’ primary evaluation.

### Measurements of IgE to penicillins (s-IgE)

S-IgE against penicillin V, penicillin G, amoxicillin (AX), and ampicillin (AMP) were measured using ImmunoCap (Thermo Fischer, Uppsala, Sweden). Results ≥ 0.35 kU/L were considered positive.

### Penicillin challenge

Patients with a present or previous positive ICT elicited by penicillin were challenged with the culprit penicillin with increasing doses: starting at 1/100 of a therapeutic dose, followed by 1/10 and finally 1/1 with 30 min intervals. Therapeutic doses were as follows: Pen V 800 mg, DX 1000 mg, MC 400 mg, AMP 500 mg, AX 750 mg, and AX + Cla 500/125 mg. For safety reasons, patients who had a delayed positive reaction to ICT (*n* = 3) or a recent anaphylactic reaction to penicillin (*n* = 2) were not challenged. Three patients refused challenge.

### Skin microdialysis

**Fig. 1:**
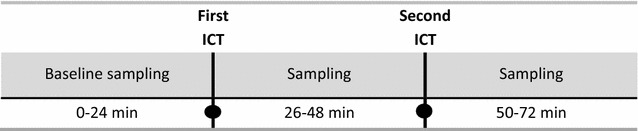
Microdialysis flow chart. The first ICT after 24 min was performed with penicillin, grass, NaCl or codeine. The second ICT after 48 min was always performed with codeine

Microdialysis is a minimally invasive technique for measuring continuous real-time release of extracellular substances. In this study, we performed microdialysis as described by Petersen et al. [18]. The microdialysis probes (EP Medical Aps^®^, Copenhagen, Denmark) were semipermeable, linear, and equipped with a guide wire. The membrane had a molecular cut-off weight of 2 kDa (outer diameter = 216 µm, wall thickness = 8 µm) allowing passive diffusion of small molecular substances. The probes were inserted intradermally and as superficially as possible into the volar forearm at a length of 2 cm of the skin using a 23G cannula. They were perfused with isotonic NaCl 0.9% at a rate of 3 µL/min. Prior to probe insertion, a local anesthetic and vasoconstrictor cream containing prilocaine and lidocaine (Emla^®^, AstraZeneca, Södertälje, Sweden) was applied to the skin for 1 h to diminish pain and bleeding from the injection sites. Emla^®^ does not affect histamine degranulation from mast cells [19]. The dialysate was sampled in glass fiber-coated microtiter wells at 2-min intervals for 72 min (Fig. 1). Depending on the number of previous positive ICTs to penicillin, the participants had 2–4 probes inserted at least two cm apart. One probe in each participant was always a control probe where first ICT was done with NaCl and second ICT with codeine.

Baseline histamine values were collected for the first 24 min after which two ICTs were performed above each probe. The first ICT was with penicillin(s) in the penicillin-allergic (culprit penicillin(s)) patients and controls, grass in grass-pollen allergics, codeine in controls (positive control), or isotonic NaCl (negative control) that were injected at a distance of 1 mm from each probe. The second ICT after 48 min was always performed by injection of codeine to collect residual histamine from the skin around the probe. When a wheal developed, it always extended across the probe. Histamine collected from the microdialysate from the 2-min sampling period was analyzed spectrofluorometrically [20]. Detection level was 5 nanogram of histamine.

Histamine release was determined in:

- 12 baseline samples
- 12 samples after first ICT
- 12 samples after second ICT

### Histamine area under curve (AUC)

As a supplement to peak histamine in wheals, the histamine under the curve, histamine AUC, was calculated by summing up the trapezoids; area = Σ((Y1 + Y2)/2*(X2 − X1)) + ((Yn + Yn − 1)/2*(Xn − Xn − 1)).The AUC comprises histaminerelease added for all 36 samples (baseline, first and second ICT).

### Statistical analysis

We analyzed data using Kruskal–Wallis test and Mann–Whitney test for ordinal data. Wilcoxon Signed Rank Test was used to determine whether ICT with penicillin induced significant release of histamine. *P* values < 0.05 were considered significant. The statistical analyses were performed with SigmaPlot 13.0, Alfasoft, Sweden.

## Results

### Microdialysis experiments on grass pollen-allergic patients and controls

**Fig. 2:**
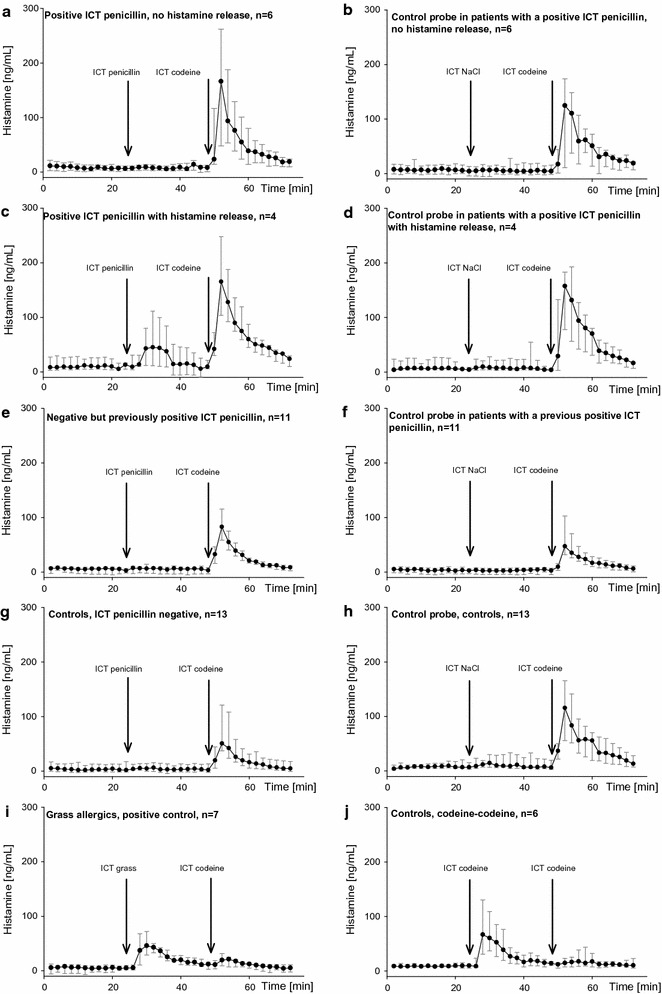
Time course of histamine release at baseline and after intracutaneous test. A 6-microliter sample of dialysate was sampled from each probe every 2 min. Results are displayed as medians and 25th and 75th percentiles

As expected, ICT with grass pollen extract induced a wheal reaction in all seven grass pollen-allergic patients (median size 13 mm, range 7.5–16.5 mm) and a significant histamine release from 2 min after first ICT (Fig. 2i). When codeine was injected at second ICT at the same site, non-significant increases in wheal size (*p* = 0.46) and peak histamine (*p* = 0.07) were observed, indicating that the grass pollen allergen released almost all the histamine from the skin mast cells. None of the controls developed wheals or released histamine after skin testing with penicillin (Fig. 2g).

When injecting codeine twice, the first codeine ICT induced, as expected, a wheal reaction (median size 11 mm, range 10.0–12.5 mm) in all six controls and a significant histamine release from 2 min after the ICT (Fig. 2j). The second codeine ICT induced a non-significant increase in wheal size in 3 of 7 controls (*p* = 0.12), and no peak histamine was observed (*p* = 0.39). We therefore use codeine 1 mg/mL as a surrogate marker for total histamine content in the skin.

### Control probe–codeine and histamine release

All penicillin patients (*n* = 21), grass pollen patients (*n* = 7) and healthy controls (*n* = 13) reacted to ICT with codeine with a wheal response showing a median diameter of 12.5 mm (range 7.5–23.5 mm) and a concomitant significant histamine release (*p* < 0.001), with a median peak histamine of 75.4 ng/mL (range 12.6–350.0 ng/mL) (Fig. 2a–h). Compared with baseline, the ICT with physiological saline gave no significant histamine release (*p* = 0.79), median peak histamine 8.6 ng/mL (range −7.6–35.5 ng/mL), and no wheals developed (Fig. 2b, d, f, h).

### Microdialysis in penicillin-allergic patients

During microdialysis, 13 positive ICTs with penicillin developed in 10 patients; one patient was positive to three different penicillins, and one patient was positive to two penicillins. Histamine was detected in four of these positive ICTs, which had median size 13.8 mm (range 12.0–20.5 mm) and a significant histamine release from 2 min after the first ICT (Fig. 2c). These positive ICTs occurred in four patients with a case history of recent anaphylactic reaction or severe urticaria to phenoxymethylpenicillin (*n* = 3) or mecillinam (*n* = 1); all had negative s-IgE (Table 1). The nine positive ICTs without histamine had a median size of 11.3 mm (range 8.5–15.0 mm) (Fig. 2a). The size did not differ significantly from the ICTs with positive histamine release (*p* = 0.17). No histamine was released in the negative ICTs in patients with a previous positive ICT to penicillin (Fig. 2e). Histamine release after ICT with codeine in patients with a positive ICT was similar for patients with a penicillin-induced histamine release (median 165.4, range 88.2–270.1 ng/mL) and patients without a penicillin-induced histamine release (median 166.2, range 5.5–308.1 ng/mL) (*p* = 0.82). Mast cells in all patients were thus able to release histamine in detectable amounts. In patients with a positive ICT to penicillin (with or without histamine release), there was a significantly higher histamine release after the second ICT with codeine compared to patients with negative ICT to penicillin, controls or grass pollen-allergic patients (*p* < 0.05) (Fig. 2a, c, e, g, i, j).

### Penicillin challenge

Intracutaneous test with the culprit penicillin was positive in only 10/21 (47.6%) of patients who previously had had a positive ICT. There was a tendency that the time interval from initial reaction until microdialysis was shorter for patients with positive ICT penicillin (median 7.5 months) than patients with negative ICT (median 27 months) (*p* = 0.084). There were no differences regarding age or gender in the two groups. In all patients, the penicillin(s) eliciting the positive ICT was identical to that causing the initial reaction.

Among the 21 penicillin-allergic patients with a previous positive ICT, 13 were deemed allergic to penicillin: 7 were challenge-positive, 2 had recent anaphylaxis, 1 had a systemic reaction to ICT, and 3 had a delayed positive ICT (Table 1). All seven patients with a positive challenge developed urticaria and/or angioedema during challenge, and all reactions were immediate i.e. commencing within 1 h after intake of penicillin. Four of the seven challenge-positive patients had become ICT negative since the initial evaluation whereas three were still positive; two of them had histamine release in the wheals. Additionally, two patients had positive s-IgE: one had negative reactions to penicillin ICT and challenge, and the other had positive reactions to penicillin ICT and challenge (but no histamine release).

One patient with a systemic reaction to ICT (Patient 3, Table 1) had participated in microdialysis six months previously, where she had been s-IgE positive and had developed a positive ICT with no histamine release (and no systemic reaction). In the planning of challenge, microdialysis was performed again and during this procedure the patient developed a systemic reaction 8 min after ICT with penicillin. Microdialysis was discontinued immediately, but the eluate already collected was analyzed and showed an increase in histamine significantly above baseline from 4 min after the first ICT (data not shown and not included in Fig. 2).

Both of the patients with recent anaphylaxis (one also IgE positive) had positive ICT with histamine release in the wheal.

A positive ICT was present in 8 of 13 patients who were deemed penicillin-allergic in this setting (Patient 1–13, Table 1), providing a sensitivity of 62%. Two of five patients with a negative challenge had positive ICT, giving a specificity of 60%. In contrast, the sensitivity of ICT with grass was 100%.

For positive ICTs with histamine release, a sensitivity of 30% and specificity of 100% were found.

### Total histamine released in the skin

**Fig. 3:**
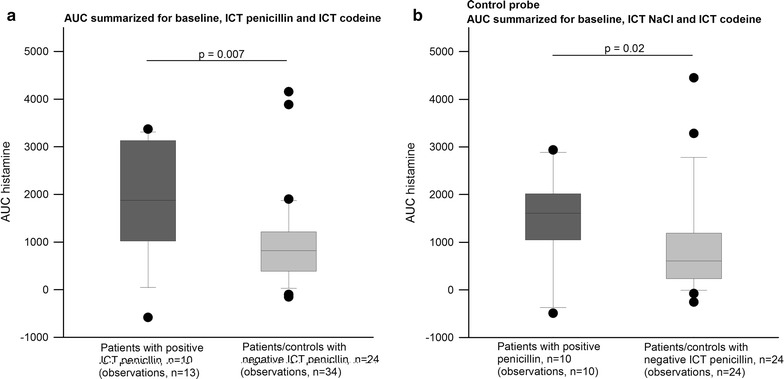
Total histamine release. Box plots showing total histamine release. **a** Significantly more total histamine release in patients with a positive ICT to penicillin compared with patients and controls with a negative ICT. **b** Demonstrates that the penicillin ICT positive patients also release significantly more histamine in total from the control probe. Results are displayed as medians, and the boundaries of the boxes represent the 25th and 75th percentiles, the whiskers above and below the boxes are the 10th and 90th percentiles. Filled circle symbolizes an outlier

There was no significant difference in total histamine release in patients with a positive ICT to penicillin with or without histamine release (*p* = 0.777), data not shown, and there was no difference in total histamine in patients with a previous positive ICT compared with controls (*p* = 0.729). Figure 3 compares all patients with a positive ICT to penicillin (with or without histamine release) and all with a negative ICT to penicillin (previously positive ICT to penicillin and controls). Patients with a positive ICT to penicillin had significantly higher histamine levels (median 1877.9 ng/mL, range 84.0–3368.2 ng/mL) than patients with a negative ICT to penicillin (median 819.4 ng/mL, range 100.7–3883.5 ng/mL) (Fig. 3a). Similar results were obtained from the negative control probe: a significantly higher total histamine release in the group with positive ICT (median 1606.1 ng/mL, range 91.2–2934.8 ng/mL) than in the group with negative ICT to penicillin (median 605.8 ng/mL, range 79.2–4448.1 ng/mL) (Fig. 3b).

## Discussion

Previously, the microdialysis technique has been applied on grass pollen allergic patients demonstrating histamine release in grass pollen induced wheals [21]. Further, it has been demonstrated that there is a correlation between size of wheal and histamine concentration in wheal [22]. To our knowledge, skin microdialysis has never been used to investigate histamine release after skin testing with an antibiotic.

All penicillin-allergic patients included in this study had a previous positive ICT to penicillin, but only 10 of 21 patients had a positive ICT to penicillin when entering the study 3–30 months after the initial positive ICT. This is in line with other studies describing declining rates of positive ICTs over time [4, 5, 23, 24]. In contrast, three patients (Patient 11–13, Table 1) showed a dual ICT response with an immediate reaction followed hours later by a delayed reaction that was persistently positive in our study, which is in agreement with data from Hjortlund et al. [17]. The fluctuating ICT response in the group of penicillin-allergic patients differs from the reproducible positive skin test to grass in the group of pollen allergic-patients with a “classical” s-IgE-mediated reaction. The difference between the two groups was further emphasized by the fact that only a few of the penicillin-allergic patients were s-IgE positive to penicillin.

A positive ICT to penicillin was only partially correlated to a positive challenge: eight ICT positive patients were deemed allergic whereas two were challenge negative. Four of the challenge positive patients had negative ICT to penicillin, but all had had positive ICT at the initial evaluation.

The four ICTs induced by histamine release all occurred in patients who were challenge-positive or had recent anaphylaxis to penicillin. Strikingly, none of these four patients had positive s-IgE, although one of the reactions was caused by mecillinam where no s-IgE is available. Interestingly, there was no detectable histamine in the ICT of any of the patients with both immediate and delayed ICT reactions nor in challenge-negative patients.

We included a control group of non-allergic healthy individuals and a control group of verified grass pollen-allergic patients. In accordance with previously published results, we found that all those with grass pollen allergy had a positive ICT to grass pollen and codeine, as well as histamine release in the wheal areas [14]. In the group of healthy individuals, only codeine caused a positive ICT and histamine release in the ICT area. These control experiments showed that the positive ICT was mediated by histamine release from the mast cells. As expected, codeine induced only a little histamine release in an ICT area previously challenged with grass pollen, indicating that the allergen caused release of almost all of the histamine from the mast cells of patients with grass pollen allergy.

When codeine was injected at the same skin site as the previously injected penicillin, all penicillin-allergic patients showed identical codeine-induced histamine release independent of a positive or negative ICT to penicillin. It might therefore be questioned whether histamine always is a key mediator in a positive penicillin-induced ICT. This is further emphasized by our finding that only 4 of 13 positive ICTs showed histamine release.

We consider codeine-induced histamine release as a surrogate marker of total mast cell histamine content in the ICT area and codeine has previously been used to evaluate total histamine in skin from patients with cold urticaria [15]. This is based on two observations. First, codeine-induced histamine release was usually higher than allergen-induced histamine release, demonstrating that codeine is a very potent histamine-releasing agent. Secondly, we found that a second injection of codeine at the same skin site induced only a marginal, and not significant, increased histamine release, indicating that most histamine was already released from mast cells by the first codeine injection.

The lack of histamine release in most of the penicillin-allergic patients during penicillin ICT points to other mechanisms than mast cell histamine release speaking against the general concept that histamine plays a pivotal role in these reactions. Other mediators such as leukotrienes [25], prostaglandins [26], platelet-activating factor (PAF) [27], bradykinin [26], or cytokines [28] might thus be upregulated in the patients’ skin. It is also possible that other cell types than mast cells are involved, and/or that non-IgE mediated mechanisms are involved such as nerve-mast cell interactions [29]. Patients with a positive ICT to penicillin released more total histamine than patients with a previous positive ICT and controls. This may indicate that patients with a positive ICT to penicillin have more mast cells in the skin, or that the histamine content in each mast cell is higher.

## Conclusion

This study demonstrates that the majority of cutaneous reactions to penicillin in penicillin-allergic patients may be caused by other mast cells mediators than histamine and may be non-IgE mediated. This contrasts with the results in patients with classic IgE mediated allergic reactions (to grass pollen), who all had near-maximum histamine release in wheals after grass pollen ICT.

In order to elucidate the complexity of penicillin-allergic reactions, future studies should be aimed at detecting other mediators in penicillin ICT wheals and search for possible non-IgE mediated allergy-like reactions.

## Abbreviations

Ana: Recent anaphylaxis
Ang: Angioedema
AX: Amoxicillin
AMP: Ampicillin
DPI: Delayed positive intracutaneous test
DX: Dicloxacillin
ENDA: European Network for Drug Allergy
ICT: Intracutaneous test
MC: Mecillinam
MDM: Minor determinant mixture
Pen G: Penicillin G
Pen V: Penicillin V
PPL: Penicilloyl poly-l-lysine
S-IgE: Specific IgE to penicillin
SPT: Skin prick test
SRI: Systemic reaction during ICT
Uns: Unspecific
Urt: Urticaria

## References

[1] Torres MJ, Blanca M, Fernandez J, Romano A, Weck A, Aberer W (2003). Diagnosis of immediate allergic reactions to beta-lactam antibiotics. Allergy.

[2] Blanca M, Romano A, Torres MJ, Fernandez J, Mayorga C, Rodriguez J (2009). Update on the evaluation of hypersensitivity reactions to betalactams. Allergy.

[3] Romano A, Blanca M, Torres MJ, Bircher A, Aberer W, Brockow K (2004). Diagnosis of nonimmediate reactions to beta-lactam antibiotics. Allergy.

[4] Tannert LK, Mortz CG, Skov PS, Bindslev-Jensen C (2017). Positive skin test or specific IgE to penicillin does not reliably predict penicillin allergy. J Allergy Clin Immunol Pract.

[5] Bourke J, Pavlos R, James I, Phillips E (2015). Improving the effectiveness of penicillin allergy de-labeling. J Allergy Clin Immunol Pract.

[6] Sogn DD, Evans R, Shepherd GM, Casale TB, Condemi J, Greenberger PA (1992). Results of the National Institute of Allergy and Infectious Diseases Collaborative Clinical Trial to test the predictive value of skin testing with major and minor penicillin derivatives in hospitalized adults. Arch Intern Med.

[7] Goldberg A, Confino-Cohen R (2008). Skin testing and oral penicillin challenge in patients with a history of remote penicillin allergy. Ann Allergy Asthma Immunol.

[8] Solley GO, Gleich GJ, Van Dellen RG (1982). Penicillin allergy: clinical experience with a battery of skin-test reagents. J Allergy Clin Immunol.

[9] Green GR, Rosenblum AH, Sweet LC (1977). Evaluation of penicillin hypersensitivity: value of clinical history and skin testing with penicilloyl-polylysine and penicillin G. A cooperative prospective study of the penicillin study group of the American Academy of Allergy. J Allergy Clin Immunol.

[10] Caubet JC, Kaiser L, Lemaitre B, Fellay B, Gervaix A, Eigenmann PA (2011). The role of penicillin in benign skin rashes in childhood: a prospective study based on drug rechallenge. J Allergy Clin Immunol.

[11] Padial A, Antunez C, Blanca-Lopez N, Fernandez TD, Cornejo-Garcia JA, Mayorga C (2008). Non-immediate reactions to beta-lactams: diagnostic value of skin testing and drug provocation test. Clin Exp Allergy.

[12] Confino-Cohen R, Rosman Y, Meir-Shafrir K, Stauber T, Lachover-Roth I, Hershko A (2017). Oral challenge without skin testing safely excludes clinically significant delayed-onset penicillin hypersensitivity. J Allergy Clin Immunol Pract.

[13] da Silva EZ, Jamur MC, Oliver C (2014). Mast cell function: a new vision of an old cell. J Histochem Cytochem.

[14] Petersen LJ, Church MK, Skov PS (1997). Histamine is released in the wheal but not the flare following challenge of human skin in vivo: a microdialysis study. Clin Exp Allergy.

[15] KringTannert L, Stahl-Skov P, Bjerremann-Jensen L, Maurer M, Bindslev-Jensen C (2012). Cold urticaria patients exhibit normal skin levels of functional mast cells and histamine after tolerance induction. Dermatology.

[16] Brockow K, Garvey LH, Aberer W, Atanaskovic-Markovic M, Barbaud A, Bilo MB (2013). Skin test concentrations for systemically administered drugs—an ENDA/EAACI Drug Allergy Interest Group position paper. Allergy.

[17] Hjortlund J, Mortz CG, Skov PS, Bindslev-Jensen C (2013). Diagnosis of penicillin allergy revisited: the value of case history, skin testing, specific IgE and prolonged challenge. Allergy.

[18] Petersen LJ (1998). Measurement of histamine release in intact human skin by microdialysis technique. Clinical and experimental findings. Dan Med Bull.

[19] Pipkorn U, Andersson M (1987). Topical dermal anaesthesia inhibits the flare but not the weal response to allergen and histamine in the skin-prick test. Clin Allergy..

[20] Skov PS, Mosbech H, Norn S, Weeke B (1985). Sensitive glass microfibre-based histamine analysis for allergy testing in washed blood cells. Results compared with conventional leukocyte histamine release assay. Allergy.

[21] Petersen LJ, Mosbech H, Skov PS (1996). Allergen-induced histamine release in intact human skin in vivo assessed by skin microdialysis technique: characterization of factors influencing histamine releasability. J Allergy Clin Immunol.

[22] Petersen LJ (1997). Quantitative measurement of extracellular histamine concentrations in intact human skin in vivo by the microdialysis technique: methodological aspects. Allergy.

[23] Blanca M, Torres MJ, Garcia JJ, Romano A, Mayorga C, de Ramon E (1999). Natural evolution of skin test sensitivity in patients allergic to beta-lactam antibiotics. J Allergy Clin Immunol.

[24] Macy E, Schatz M, Lin C, Poon KY (2009). The falling rate of positive penicillin skin tests from 1995 to 2007. Perm J..

[25] Soter NA, Lewis RA, Corey EJ, Austen KF (1983). Local effects of synthetic leukotrienes (LTC4, LTD4, LTE4, and LTB4) in human skin. J Invest Dermatol.

[26] Wallengren J, Hakanson R (1992). Effects of capsaicin, bradykinin and prostaglandin E2 in the human skin. Br J Dermatol..

[27] Basran GS, Page CP, Paul W, Morley J (1982). Cromoglycate (DSCG) inhibits responses to platelet-activating factor (PAF-acether) in man: an alternative mode of action for DSCG in asthma?. Eur J Pharmacol.

[28] Petersen LJ, Brasso K, Pryds M, Skov PS (1996). Histamine release in intact human skin by monocyte chemoattractant factor-1, RANTES, macrophage inflammatory protein-1 alpha, stem cell factor, anti-IgE, and codeine as determined by an ex vivo skin microdialysis technique. J Allergy Clin Immunol.

[29] Foreman JC (1987). Neuropeptides and the pathogenesis of allergy. Allergy.

